# Alcohol as a Medicine

**Published:** 1872-06

**Authors:** 


### ALCOHOL AS A MEDICINE

Has merits similar to the various preparations of opium; it often gives time in cases of emergency, it supports and sustains for the moment; for a moment it arrests the running down of the system, revives the waning powers of life, and thus affords an opportunity to employ measures which have an enduring effect. Of itself, it has no curative power whatever; by no possibility does it ever impart one atom of strength to the system. Although it may seem to do so, in many cases, it really does not, any more than the lash on the beast of burden, fallen under the heavy load, makes that beast rise from the earth again by imparting new strength! It calls for that much additional strength in advance, only the next moment to leave the beast weaker than it was before the lash was applied, by just as much as the summoned strength required to enable it to spring up again under the goading. A man may have ten dollars a day allowed to support him; but if to-day he draws for it, as well as for to-morrow's payment, he is just that much poorer to-morrow than if he had not drawn its money beforehand.

Alcohol is important as a means of withdrawing from barks, and roots, and leaves, certain medicinal qualities which could not be obtained by soaking in water or by boiling; but to use it as possessing distinctive medicinal virtues of itself, is not only a fallacy, but it is a positive, demonstrated evil under all circumstances.

The more we take of ordinary drugs, the more repulsive do they become, until the repetition amounts to a torture. Any remedy combining alcohol does, by virtue of the alcohol, beget a relish, a taste, a liking, a longing for its use, increasing constantly, requiring it oftener and in larger quantities, until, before the patient is aware of it, he begins to feel the want of it, and finally cannot do without it, as shown in the following illustration:—

### THE BOOK AUCTION.

It was a famous library. Its former owner, a man of great wealth and greater fame, had died. He had spared no expense necessary to get any book he wanted; and when it was found out that it was to be sold, people came a thousand miles to purchase some of the volumes. The former owner never expected that these choice treasures of his, collected with so much care, so highly valued, so dearly loved by him, should fall to the fate of a common auction; but they did, and in a way which should burn a lesson into every young man's heart deep down to its very core, so that it could never, by any possibility, be forgotten for a single moment of existence in connection with the circumstances of the case.

The great man was a bachelor; he had a sister whom he dearly loved, and she was dead, leaving a son, a young man in the very prime of life. In memory of that dead sister he left his library to that son, who unfortunately had fallen into drinking habits, as a possible means of reformation. The deceased ordered that this library should be transferred to his nephew, his loved sister's son, if he would not drink spirits or malt liquors for five years. Half the time had not passed before the young man forfeited his rights by the will, and the library was sold at auction yesterday for over thirty-one thousand dollars, proving but too powerfully the terrible

TYRANNY OF DRINK.

Here was a young man who was offered six thousand dollars a year for five years if he would not drink liquor, but it really seems as if he would rather

DRINK AND DIE

than to curb his passion for the intoxicating cup. What have fathers and mothers to say to all this, when they have made toddy for their children; and young ladies, who invite their New Year's callers to take a glass of wine; and bankers and business men, who allow their clerks to see them indulging in "treats" to one another; and men who make these liquors to sell, and men who sell them, — what have you all to say to a lesson like this, if it were your own brother, your own son, to whom somebody else had sold liquor; for whom somebody else had made it; which some young lady's brother had been invited to drink at a New

Year's call? The beautiful wine, the sparkling champagne, the warming cordial, the foaming cup, all have it said of them at last,—

"IT BITETH LIKE A SERPENT AND STINGETH LIKE AN ADDER!"

It is not the least evil of spirituous liquors that, when persons get well by taking alcoholic medicines, they are very often unable to abandon their use; hence it is as demonstrable as any mathematical problem, that its employment as a medicine for its own sake, and for its own merits, is irrational and unwise, because it is murderous.

Some years ago, a young physician of splendid talents adopted a theory, and endeavored to sustain it before a London public, that alcohol had a curative power in inflammations as well as fevers; and that in typhus and typhoid it was the sheet anchor. For a short time, brandy in all its forms became the rage, until the more conservative of the medical faculty thought of calculating the results, when it soon appeared that Dr. Todd lost more patients than any other man in London. He practiced his system on his own person, and died but a little over forty years of age.

In two years, in a London hospital, eight per cent., eight persons out of every hundred, died among the medical cases where alcohol was freely used among the patients; among the surgical cases, five in a hundred died, which was an increase of fifty per cent. of deaths taking place in the same hospital just before, when alcohol as a medicine was not allowed. In various experiments made in England, with a view to settle the question as to hospital practice, there never was a failure in the end, whether in town or city or country, to show the fact that the more liquor was used in the medicines, the larger was the proportion in deaths.

Dr. Mussey, of Cincinnati, Ohio, who was second to none in his day as a surgeon and physician, personally known and consulted by the writer, has left on record this suggestive statement:—

"Trains of morbid symptoms are often aggravated by a stimulant practice, and new centres of irritation are established, which, if not sufficient to destroy the strength of the patient, prolong the period of fever, and frequently cause relapses or a lingering convalescence."

In large cities, where great stakes are played for at the stock board and on 'change, where men's wits and ambitions are daily under the very strongest tensions that human motives can generate, large and frequent draughts are made upon the decanter and the brandy bottle; scores of men are now to be seen any day in Wall Street and around the doors of the Stock Exchange who are known to be always "full," and never empty of alcohol in some form. Such men are constantly dying without a moment's warning; glibly stated in the newspaper, "Died suddenly, yesterday, of

"HEART DISEASE,"

when in reality it was because the man had kept himself up, to the very last moment, by the more and more unstinted use of brandy, until outraged nature refused to be goaded longer, and the thread of life was snapped forever!

"I CAUTION YOU,"

said a distinguished physician to a graduating class of young doctors, "against employing wine as a substitute for the true restorative treatment." He had himself largely used alcoholic preparations, until startled one day by a fact which suddenly stared him in the face, that twenty per cent. of his patients died. He immediately reversed his system, discarded alcohol altogether, and lost less than three in a hundred.

Two eminent names in medicine in China and in Italy lost seven per cent. of their fever patients, whereas previously, under the use of alcohol, they lost twenty-eight in a hundred, instead of seven, without its use.

Prince Albert, the husband of Queen Victoria, was taken ill of typhoid fever in consequence of an opening in an old sewer or house drain, by which the foul air escaped into the prince's library, where he spent most of his time. Typhoid fever is one of debility; all the powers of life are oppressed; body and mind seem to labor under a load from which they cannot escape. There is not a malady known in which it would not seem natural and reasonable that if stimulants were given, the effect would be to rally the patient, to lift him up, and give a power to overcome disease. This was the prevailing view at the time among educated medical men, and that practice was adopted, the prince himself strongly objecting to it, and only submitting from a sense of duty to others. This at least is the received statement. He died. The same result might have occurred had no alcohol been taken. It certainly did not save; and it may well be concluded that if it did no good in a case where it would seem it was the most appropriate of every human remedy, it ought not to be considered a medicine, curative of anything, but utterly worthless; and it is the world's misfortune that it should have been relied on in the case of so exalted a personage, whose private and public character commanded the high respect of the civilized world.

Alcohol is not only not efficient in the cure of diseases in which its use would seem to be peculiarly beneficial, that is, in diseases of debility; but there are ailments curable by other means, which become fatal by its employment. One of these is what is known as

RHEUMATIC FEVER, which, in numbers of cases in the practice of eminent men, has been driven to the heart by the employment of alcoholic remedies. It was the common practice some years ago to treat this malady by stimulation. Dr. Reeve was one of the first to draw attention to the frequency of heart disease as following rheumatic fever when treated with alcohol. On abandoning its use, this fever did not run into heart disease, as formerly; only one in a hundred. Five sixths of all the cases of rheumatic fever attended by the brilliant advocate of stimulant treatment, the late Dr. Todd of England, terminated in heart disease, — a result fearful to contemplate. Yet the doctor was so enthusiastic in the support of his theory, that the fatal results of his practice did not arrest his attention until the statistics were brought to his notice by his medical brethren who held other views.

A celebrated physician once said, "Alcoholic stimulants are a two-edged sword in the hands of the practitioner;" and so it appears from the observation and experience of Dr. Gardner, Professor of Medicine in the University of Glasgow. In six hundred cases of typhus fever treated in the hospital there, the recoveries increased in the direct proportion in which milk and buttermilk were used to "support" the patient, instead of alcohol; when two and a half ounces of wine a day were allowed each patient, instead of thirty-four ounces, which is a little over a quart, the mortality diminished more than one fourth, more than twenty-five per cent.

Of over two hundred cases of typhus fever treated without alcohol, in the Public Infirmary of Glasgow, not one died; in another institution, where wine was relied on, there were many deaths. All know the harmful effects of

BAD BLOOD.

It is the direct cause of a large class of diseases. The incalculable benefit of pure air in sickness and in health, as a means of purifying the blood, of making it "good," instead of "bad," is universally acknowledged. In fact, cleanliness, warmth, and pure air would cure a multitude of cases of sickness; but the first, the direct, the infallible effect of taking alcohol into the stomach, is to prevent the blood from being unloaded of its carbonic acid, the most deadly of constituents, so much so that when a small proportion of it is found in the air we breathe, we fall senseless to the earth, as with the rapidity of lightning.

The air we breathe is made of oxygen and nitrogen; it is the former which gives us life, which imparts to the blood its sparkling qualities, its healthful activities; but alcohol immediately attracts this oxygen into it; absorbing it, appropriating it to its own use; becoming commingled with it; hence it cannot go to the blood, and the blood cannot be purified.

Alcohol goes to the lungs the instant it is swallowed; we perceive it in the breath. The air we breathe goes also to the lungs; and it is there and then that it is robbed of its life-giving power by this

GREAT ENEMY OF MAN, alcohol, in whatever shape found, or in whatever combination, whether in brandy, whiskey, rum, gin, wine, cider, porter, ale, or beer. When it is taken into account that in the treatment of all sickness a full supply of oxgyen for the blood is to be derived from the air breathed, and that alcohol, as just explained, appropriates the oxygen of this air to itself at the very fountain-head, not only robs the air of its purity, but prevents the blood at the same time from being unloaded of its poisonous quality, its carbonic acid, it becomes a demonstration that in no common sickness is alcohol beneficial; that in every human disease it becomes a bane, a positive harm. Just as well may we expect a sick man to get well without pure air, as for him to get well by using spirits; hence, in sickness, no form of alcohol ever does any real, permanent good, but always does do harm.

Alcohol in the form of toddies, or beer, porter, ale, and cordials, is taken to a great extent by ignorant mothers to

MAKE MILK FOR THE BABY, but it is well known that if a mother drinks spirits, and nurses her infant a short time afterwards, that infant feels the effects of the drink; and as the mother soon finds herself needing the drink, — at least feels the loss of it, — and the appetite craves it, so a craving is begotten in the infant for the stimulant which the mother pines for, to be exhibited in after life by an

UNACCOUNTABLE HANKERING

for drink, next, a yielding to it; and a parent finds his child a drunkard, a confirmed drunkard, before twenty years of age. It is terrible to think of. Everybody is surprised that the child of such a person should turn out a drunkard, little dreaming that it was made a drunkard on its mother's knee.

A few years ago, in New York city, there was a perfect rage for

WHISKEY IN CONSUMPTION.

There was an establishment in Bond Street where whiskey was breathed into the lungs, poured into the stomach, and fairly rubbed into the skin. Men and women crowded to the portals of that temple for health, and the delusion did not vanish until the chief man himself died in a drunken debauch.

When a man has consumption his lungs are always in a condition which makes him

SHORT OF BREATH.

This is a symptom which is never by any possibility absent, as proven by its being brought on in ascending a flight of stairs even slowly, or in walking a little fast. This shortness of breath is always the result of one or two causes: either part of the lungs are gone, have decayed away, or they are so filled up with matter or phlegm, or more solid substances, as tuberculous deposits, that enough air cannot get in to supply the wants of the system; that is, it cannot get oxygen enough; but if we drink whiskey, it begins at once to absorb the oxygen, thus diminishing the already deficient supply, and directly advancing the progress of the disease, destroying the chances of recovery, and making a fatal termination more speedy and more certain.

In the treatment of over two hundred cases of consumption, in various stages, a distinguished practitioner says: "One third of these began while the persons were habitually using liquor, and had been from one to ten years."

It would be difficult to obtain from any eminent physician a verified statement that he had ever seen a single case of real, permanent improvement in a consumptive patient from the use of spirits. There is, in many cases, an apparent improvement, but it is uniformly followed in a very few weeks by a deranged digestion, a diminished appetite, a more rapid falling away, and a more utter prostration. As many drunken men and women have been led into the habit by having obtained a taste for spirits in using medicines containing alcohol in the shape of wines and brandies, the thoughtful and conscientious reader will feel warned against so dangerous a mistake, while every mother with a true mother's heart will fear to nurse her babe into habits of drunkenness by using spirits in any form, for any cause, at any time.

